# Utility of the Rowland Universal Dementia Assessment Scale and INECO Frontal Screening for differentiating dementia subtypes between Alzheimer's disease and Parkinson's disease dementia

**DOI:** 10.1177/25424823251335193

**Published:** 2025-04-17

**Authors:** Gregory Brown, Diego Bustamante-Paytan, María Fe Albujar Pereira, Jose Huilca, Katherine Agüero, Graciet Verastegui, Zadith Yauri, Rosa Montesinos, Nilton Custodio

**Affiliations:** 1 Unidad de Investigación, Instituto Peruano de Neurociencias, Lince, Peru; 2 Department of Neurology, University of California, San Francisco, CA, USA; 3 Centro de Investigación del Envejecimiento, Universidad de San Martín de Porres, La Molina, Peru

**Keywords:** Alzheimer's disease, dementia, diagnosis, global health, neuropsychological tests, Parkinson's disease

## Abstract

**Background:**

Time to dementia diagnosis is a major barrier to effective care, particularly in resource-limited settings such as Latin America. Barriers to timely dementia diagnosis include the lack of access to comprehensive neuropsychological testing, cognitive specialists, and advanced diagnostic tools. Brief cognitive assessments, such as the Rowland Universal Dementia Assessment Scale (RUDAS) and INECO Frontal Screening (IFS) offer promise for diverse populations, and may help in specific dementia subtypes, including Alzheimer's disease (AD) and Parkinson's disease dementia (PDD).

**Objective:**

This study evaluates the efficacy of RUDAS and IFS in comparison to the Mini-Mental Status Exam (MMSE).

**Methods:**

A total of 243 participants (70 normal cognition Controls, 62 PD with normal cognition, 46 PDD, and 75 AD) were recruited as part of an observational cross-sectional study at a cognitive clinic in Peru. Diagnosis was based on clinical criteria and confirmed with a comprehensive neuropsychological battery. Participants underwent cognitive assessment using RUDAS, IFS, and MMSE.

**Results:**

Both RUDAS and IFS differentiated dementia from normal cognition groups with 100% specificity, compared to 53% for MMSE. The IFS identified early cognitive changes in PD (median score: PD = 24; Controls = 27, p < 0.001). RUDAS was particularly effective in distinguishing AD and PDD using the memory and visuospatial tasks.

**Conclusions:**

These results suggest that RUDAS and IFS can enable faster and clearer diagnoses for dementia subtypes, offering clinicians and community health workers practical tools to improve care in resource-limited settings where comprehensive evaluations are not always feasible.

## Introduction

Dementia represents a growing public health challenge, particularly in resource limited areas, such as Latin America. Delayed diagnosis has been identified as a major barrier to effective care,^1^ with many older adults facing challenges, such as insufficient specialty care, lack of advanced diagnostics tools (i.e., positron emission tomography scans or blood/cerebrospinal fluid biomarkers), socioeconomic constraints, confusing healthcare systems, and lack of transportation infrastructure. Therefore, the ability to detect cognitive deterioration and dementia are largely unmet,^2–4^ Latin American governments have identified training professionals in the recognition of dementias and improving access to diagnosis and care as major principles for improving dementia care policies.^5^ Understanding the distinct neurodegenerative processes involved in Alzheimer's disease (AD) and AD-related dementias, such as Parkinson's disease dementia (PDD) is crucial for advancing clinical practice and improving patient outcomes. Tools that enable faster and clearer diagnoses are needed to guide clinical practice and improve patient care.

Brief cognitive assessments provide a valuable tool for allocating and directing support resources, which has been identified as one of the most important aspects of dementia care, accounting for over 50% of time spent on care.^6^ Ideally, these tests should be useful across various settings, populations, and cultural biases. However, commonly used assessments, such as the Mini-Mental Status Exam (MMSE), are limited by biases regarding language, culture, and ethnicity.^7^ To combat these issues, the Rowland Universal Dementia Assessment Scale (RUDAS) has emerged as a multi-culturally sensitive cognitive assessment that is not affected by language and education. RUDAS has been validated in over 20 countries, including Peru,^8^ Chile,^9^ and Brasil,^10^ demonstrating consistent specificity and sensitivity across high- and low-income settings.^11^ However, the utility in PD, the second most common neurodegenerative condition^12^ and a prevalent cause of dementia,^13,14^ remains poorly understood. Use of the MMSE has been discouraged in PD,^15^ so an effective brief cognitive assessment remains of vital importance. The INECO Frontal Screening (IFS) has demonstrated potential in Peru^16^ and other countries,^17,18^ and may hold specific promise in PD, due to the characteristic presence of early frontal executive and attentional deficits.^19^ However, the IFS has only been minimally investigated in PD and PDD,^20^ leaving a critical gap for its validation in this population.

In this study, we examine the performance of RUDAS and IFS across four patient groups: Control, PD, PDD, and AD, and compare these results with those from the MMSE. We hypothesized that IFS would effectively detect PD-specific cognitive changes, while RUDAS memory task performance could help different AD from PDD. These findings aim to improve early diagnosis and dementia subtype classification, enhancing care, especially in resource-limited settings.

## Methods

### Study design and participants

This observational cross-sectional study included 243 participants recruited consecutively from the Peruvian Institute of Neurosciences between June 2019 and May 2024. Participants were divided into four groups: 70 cognitively normal controls, 62 PD patients with normal cognition, 46 PDD patients, and 75 AD patients. Inclusion criteria required participants to have either normal cognition or a diagnosis of PD, PDD, or AD, based on established clinical criteria.^21–24^ All participants were evaluated by neurologists and neuropsychologists with expertise in dementia and underwent brain magnetic resonance imaging (MRI) using a standardized protocol to assist with diagnosis and exclude structural abnormalities. Exclusion criteria included history of head trauma, active epilepsy, prior stroke, inability to undergo MRI, and abnormal MRI findings suggesting brain tumors or infarcts.

### Neurocognitive assessment

#### RUDAS

The RUDAS can be administered within 10 min and comprises of 6 sections with a maximum of 30 points: memory (8 points), visuospatial orientation (5 points), motor praxis (2 points), visuospatial construction (3 points), judgment (4 points) and semantic fluency (8 points). The RUDAS has a maximum score of 30 where a lower score denotes poor cognitive performance.^25^ Several studies have been published validating the RUDAS among Peruvians with a middle-education level from an urban area of Peru^26^ and urban^27^ and rural^8^ Peruvians with illiteracy. The most common cutoff for RUDAS is <22, with reported sensitivity ranging from 0.78 to 0.86 and specificity from 0.78 to 0.87.^11^

#### IFS

The IFS test evaluates executive functions taking roughly 10 min to conduct and comprises 8 sections with a maximum of 30 points: motor programming (3 points), conflicting instructions (3 points), motor inhibitory control (3 points), backward digital span (6 points), verbal working memory (2 points), spatial working memory (4 points), abstraction (3 points), and verbal inhibitory control (6 points). A Peruvian version of IFS was found to have a sensitivity of 94.12% and specificity of 94.2% at a cutoff of <18 points.^16^

#### MMSE

The MMSE takes 5–10 min to administer and comprises 5 sections with a maximum of 30 points: orientation (10 points), registration (3 points), attention and calculation (5 points), recall (3 points), and language and praxis (9 points). A Peruvian version was adapted and validated (modified from the Argentinian version)^28^ incorporating cultural modifications specific to Peru. Cutoff for dementia screening has been debated in MMSE, but <25 is a common threshold.^29^

#### Neuropsychological battery

We used the neuropsychological battery Uniform Data Set of the from the National Alzheimer's Coordinating Center (UDS, NB 3. 0).^30^ This included immediate and delayed recall tasks (Craft Story 21, Benson complex figure), the Multilingual Naming Test (MINT), number span tests (forward and backward), categorical fluency (animals and vegetables), verbal fluency (P and M letters), and the Trail Making Test A and B.

### Clinical classification

**Figure 1.**
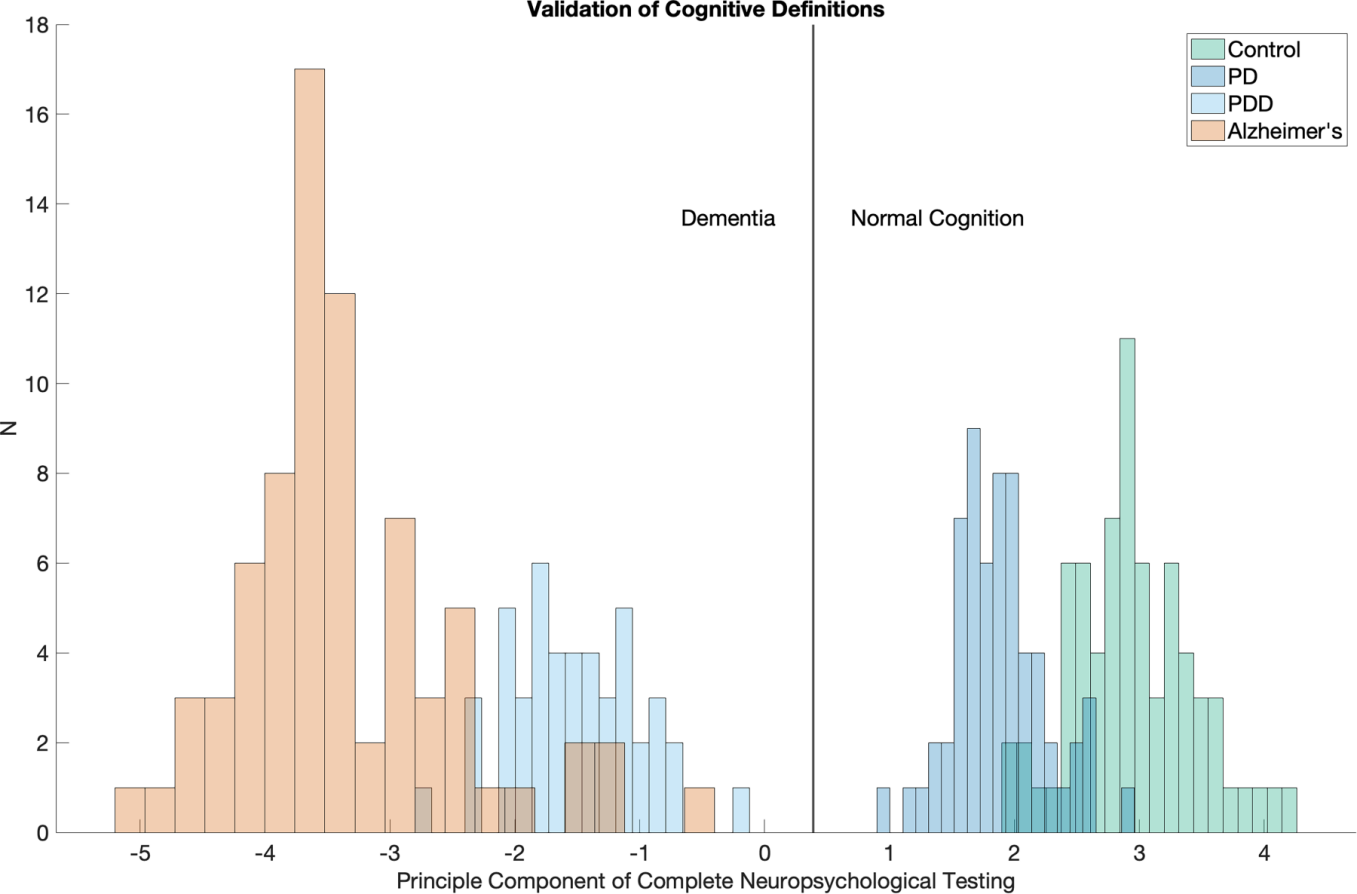
Validation of cognitive definitions. Histogram results from the first principal component of the full neuropsychological testing: forward digit span, backward digit span, Craft21 immediate recall, Craft21 delayed recall, Benson complex figure immediate recall, Benson complex figure delayed recall, multilingual naming test, trails making A, trails making B, Semantic Fluency (Animals), Phonological Fluency (Letter P). The principal component was well balanced across all tests and clearly differentiated cognitively normal and dementia groups.

Dementia diagnosis was established through comprehensive clinical evaluation by neurologists specializing in dementia, following standardized criteria.^21–24^ Cognitive assessment was conducted using the UDS, NB 3.0, as described above.^30^ Trained neurologists or neuropsychologists administered the battery to ensure consistency and reliability. All patients underwent MRI to exclude alternative neurological causes of cognitive impairment. Additionally, depression screening with Beck's depression index (BDI) was performed to rule out major depressive disorder as a potential confounder. Dementia was defined as a Clinical Dementia Rating (CDR) score of 1 or higher, based on information obtained from both study participants and their caregivers/companions. In cases of diagnostic uncertainty, final classification was determined by a consensus panel consisting of neurologists, geriatricians, neurorehabilitation specialists, and neuropsychologists. Accurate differentiation between cognitively normal and dementia was confirmed using the first principal component of the full-neuropsychological testing from the UDS dataset (Figure 1).

Parkinson's disease diagnosis for both the PD and PDD groups was based on the United Kingdom Parkinson Disease Society Brain Bank listed criteria.^31^ PD participants were excluded if motor symptoms did not proceed cognitive symptoms by at least 1 year (the 1-year rule).^24^ Cases with diagnostic uncertainty were resolved by consensus among neurologists, geriatricians, neuro-rehabilitation specialists, and neuropsychologists.

### Statistical analysis

All analyses were conducted in MATLAB. Demographic data were compared using the Kruskal-Wallis test for continuous variables (age, education, BDI, disease duration, UPDRS) and the chi-squared test for categorical variables (sex, H & Y score). If significant differences among the four groups were found, subgroup analyses were performed with Bonferroni correction for multiple comparisons. A k-means approach was employed to identify cognitive test cutoffs separating dementia from normal cognition. Mean and standard error of the mean were presented in bar graphs for subtest comparisons. Significance (ɑ < 0.05) was determined using the Kruskal-Wallis test, adjusted for age, sex, and education via linear regression. Additionally, a factor analysis based on linear discriminant analysis loadings identified the subtest combination that best discriminated PDD from AD.

### Standard protocol approvals, registrations, and patient consents

The research activities involved in this study have been conducted in accordance with the ethical standards of the Helsinki Declaration. The study was approved by the Committee for medical and health research ethics, Hospital Nacional Docente Madre-Niño-HONADOMANI “San Bartolomé” (10360-18). All participants participated voluntarily in the study and provided written informed consent.

## Results

### Demographics

**Figure 2.**
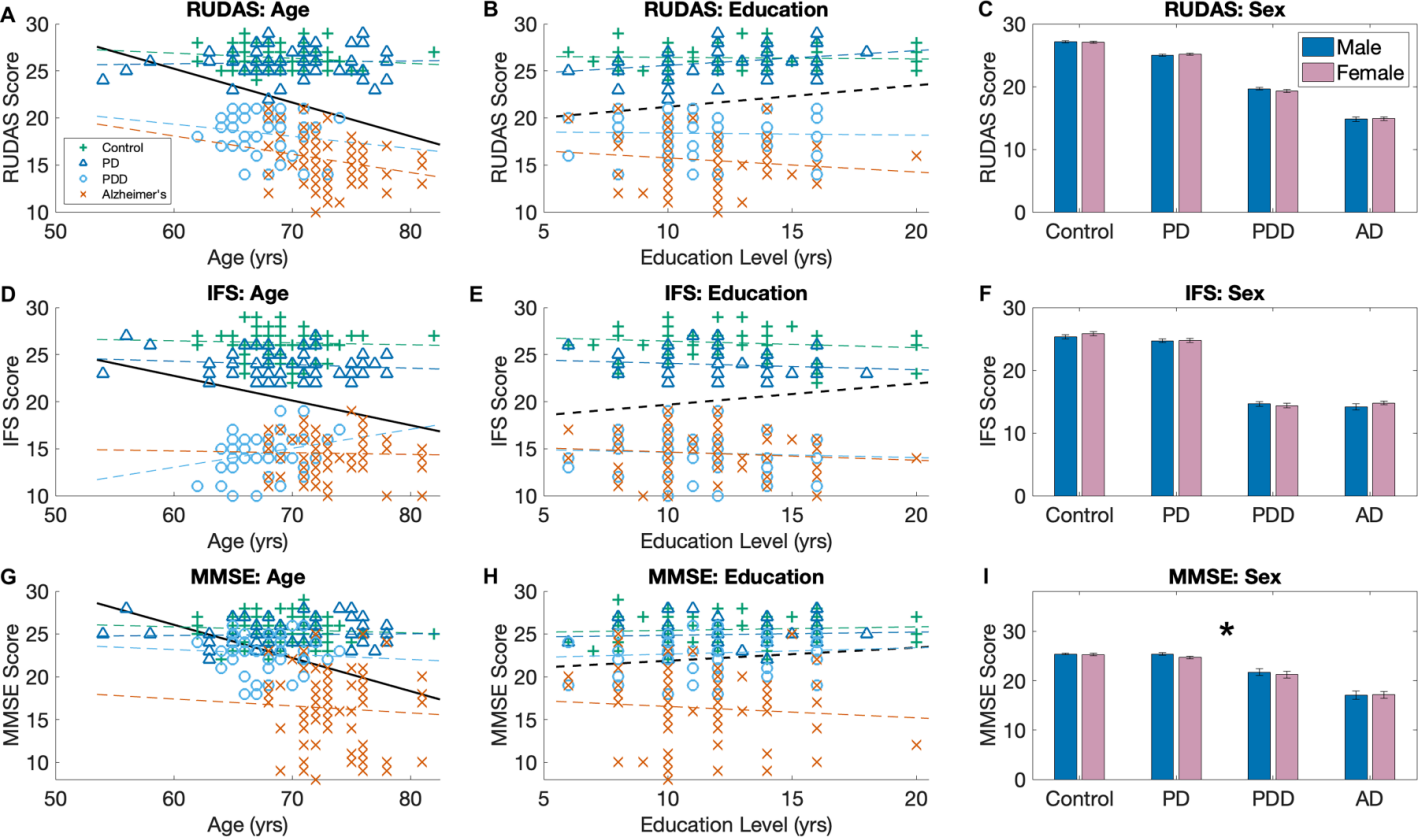
Associations with demographics. Associations between brief cognitive tests and age (a, d, g), Education (b, e, h), and Sex (c, f, i). Colored lines represent the best fit for the specific group. Dark black line represents the best fit for all participants. Solid line indicates significant correlation (p > 0.5). For sex, only MMSE scores were significantly different between males and females when considering all patients. There were no significant differences between the individual groups (using a chi-squared test).

We recruited 253 participants across the four groups: 70 control, 62 participants with PD and normal cognition, 46 participants with PDD, and 75 participants with AD. All data was complete. The AD participants were significantly older than the other groups (p < 0.001), while PDD participants were younger (p < 0.004). Age was significantly associated with cognitive performance when analyzing all participants together, but no significant correlations were found within individual diagnostic groups (Figure 2). Most participants (96%) were between 60–80 years of age. No significant group differences were observed in sex or education level (Table 1), and these factors were not associated with cognitive performance (Figure 2).

**Table 1.** Demographics.

	Control	PD	PDD	AD	p
N	70	62	46	75	
Age	69 ± 3.4	69.8 ± 5.4	67.5 ± 2.7	73.2 ± 3.1	C.D.E.F.
Sex (%F)	39%	31%	22%	40%	N.S.
Education	12.3 ± 3.2	12 ± 2.4	11.2 ± 2.7	11.5 ± 2.5	N.S.
BDI	5.9 ± 2.7	7.8 ± 2.3	6 ± 3	7.4 ± 2.8	A.C.D.
Disease Duration	–	4.8 ± 1.2	5.5 ± 1.3	–	D
UPDRS III	–	33.9 ± 7.4	29.4 ± 11.4	–	D
H & Y Score (%)					N.S.
1	–	0%	0%	–	
2	–	73%	70%	–	
3	–	27%	30%	–	
4	–	0%	0%	–	
5	–	0%	0%	–	

Demographic data for the four groups. For continuous variables (age, education, BDI, disease duration, and UPDRS), data are presented as mean ± standard deviation. Analysis was first performed across all 4 groups using the Kruskal-Wallis test. If this was significant, a sub-analysis was performed to identify the significant comparisons with letters indicating the significance of these comparisons: A: Control versus PD; B: Control versus PDD; C: Control versus AD; D: PD versus PDD; E: PD versus AD; F: PDD versus AD; N.S. indicates no significance at the group level. For categorical variables (Sex and H & Y score), data are presented as percentage of group. A chi square test was used with a similar convention. AD: Alzheimer's disease; BDI: Beck's Depression Index; F: female; H & Y score: Hoehn and Yahr Score; N: group count; PD: Parkinson's disease; PDD: Parkinson's disease dementia; UPDRS III: Unified Parkinson's disease Rating Scale-III (motor component).

Depression scores were higher in the AD and PD groups; however, the averages were in the no depression range (BDI score <10), and no participant met the threshold for moderate depression (BDI score ≥ 19). For the PD and PDD groups, PDD had a longer disease duration and milder motor symptoms. All PD and PDD participants were classified in Hoehn and Yahr (H&Y) stages 2–3, with no differences in H&Y staging across groups.

### Total scores

**Figure 3.**
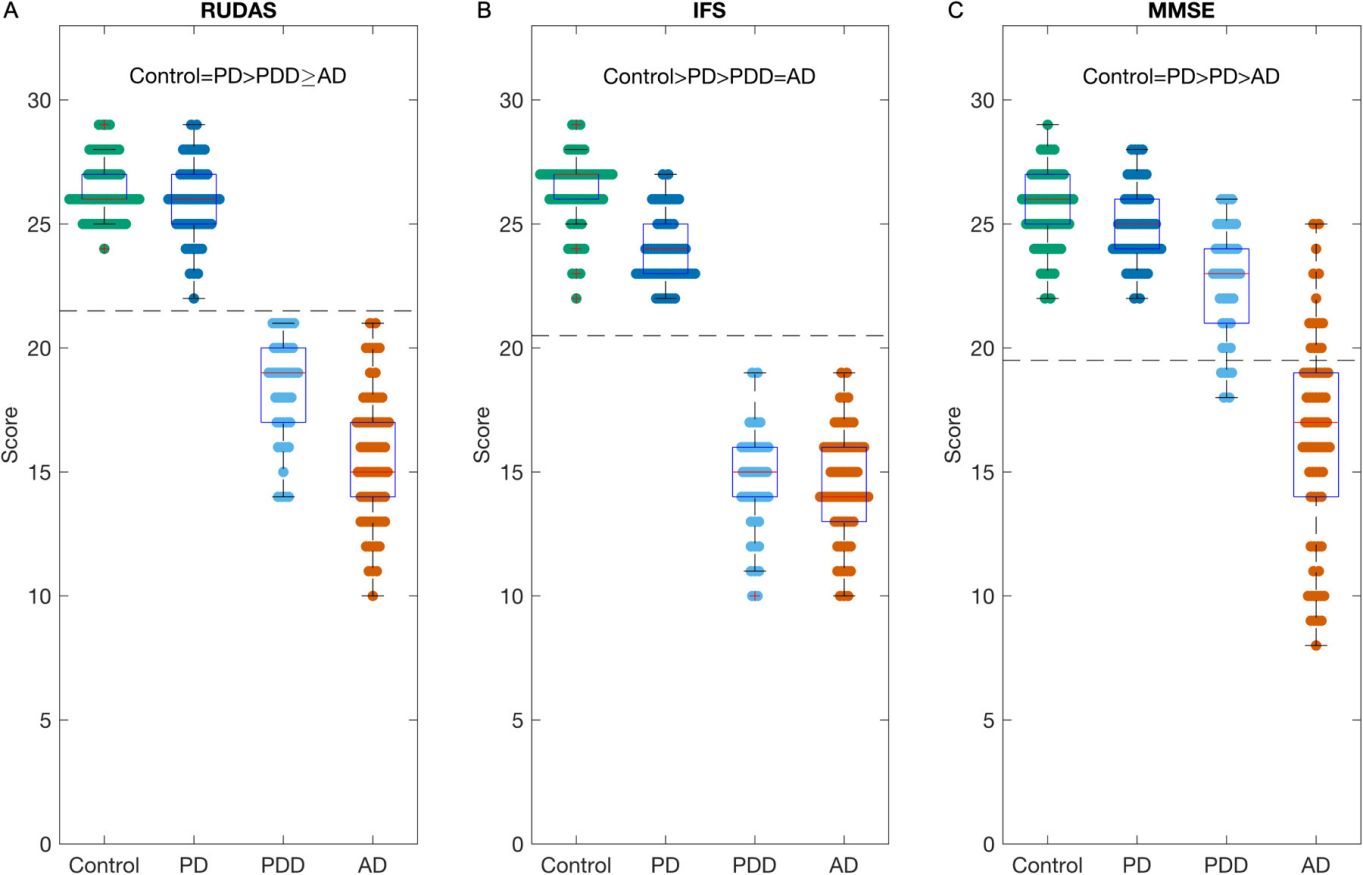
Boxplots of brief cognitive tests. Results for the each of the 3 brief cognitive tests (a) Rowland Universal Dementia Assessment Scale (RUDAS), (b) Frontal Screening (IFS), and (c) Mini-Mental Status Exam (MMSE). The data is presented for the four groups: Control, Parkinson's Disease (PD), Parkinson's disease dementia (PDD), and Alzheimer's disease (AD). > indicates significant difference between groups after correction for correcting for age, sex, and education. ≥ indicates that the significant difference does not withstand correction for correcting for age, sex, and education. The dotted line represents the optimal dementia cutoff. RUDAS: <22, IFS: <21, and MMSE: <20.

For RUDAS scores, we found Control = PD > PDD > AD (Figure 3(a)). After adjusting for age, sex, and education, the PDD > AD association was no longer significant. Both normal cognition groups had a median of 26. For the dementia groups, the range was 14–21 with a median of 19 for the PDD group and 10–21 with a median of 15 for the AD group. Therefore, a cutoff of ≤21 had 100% specificity and sensitivity for differentiating the dementia from normal cognition.

For IFS scores, we found Control > PD > PDD = AD, and these associations remained significant after correcting for age, sex, and education (Figure 3(b)). IFS scores ranged from 22–29 with a median of 27 for the Control group and 22–27 with a median of 24 for the PD group (p < 0.001). For the dementia groups, the range was 10–19 for both the PDD and AD groups, and the medians were 15 and 14 respectively. A cutoff of ≤21 had 100% sensitivity and specificity for differentiating dementia from normal cognition.

For MMSE scores, we found Control = PD > PDD > AD, and these associations remained significant after correcting for age, sex, and education (Figure 3(c)). We found MMSE was less effective in differentiating the groups. For the control group the range was 22–29 with a median of 26 and for the PD group the range was 22–28 with a median of 25. For the dementia groups, the MMSE range was 18–26 with a median of 23 for the PDD group and 8–25 with a median of 17 for the AD group. Using a cutoff of <20, we found a sensitivity of 1, but a specificity of only 0.55.

Due to the associations of brief cognitive assessments with age, a sub-analysis was performed by correcting the test scores for age and attempting to classify dementia (PDD and AD) from normal cognition (healthy controls and PD without dementia) (Supplemental Figure 1). After age correction, RUDAS achieved an accuracy of 98% (sensitivity: 99%, specificity: 97%), IFS had an accuracy of 99% (sensitivity: 98%, specificity: 99%), and MMSE demonstrated an accuracy of 81% (sensitivity: 71%, specificity: 91%).

### Subtests

**Figure 4.**
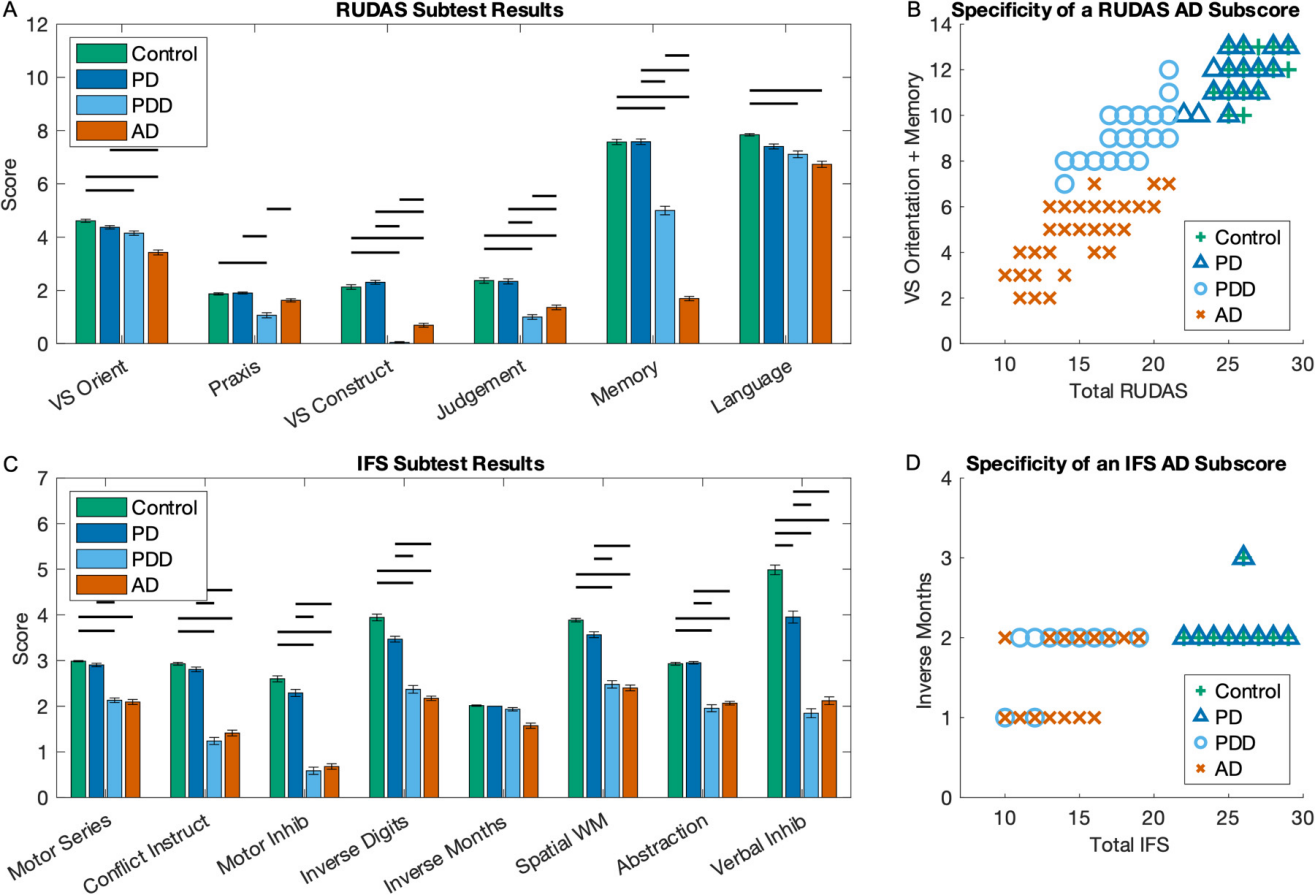
Results of sub-tests. The results for individual subtests for the (a) Rowland Universal Dementia Assessment Scale (RUDAS) and (c) INECO Frontal Screening (IFS). Vertical bars represent standard error of mean. Horizontal bars represent significance (p < 0.05), after correcting for age, sex, education, and multiple comparisons. (b) Displays the data using a possible “Alzheimer's disease (AD) subscore”, which is the sum of the visuospatial (VS) orientation and memory parts. Of note, multiple participants may occupy the same data point space. (d) In contrast to the RUDAS, a potential “AD subscore” in IFS is much less effective and only includes the inverse months task.

Investigating RUDAS subtests, no significant differences were observed between the Control and PD groups (Figure 4(a)). Scores on visuoconstruction, judgment and memory were lower in both dementia groups compared to both normal cognition groups. The AD group scored lower on the memory task compared to the PDD group, while the PDD scored lower on praxis, visuoconstruction, and judgment tasks compared to the AD group. Praxis scores were lower in the PDD group, and visuospatial orientation scores were lower in the AD group compared to both normal cognition groups, while visuospatial orientation was only lower in PDD compared to the control group, not the PD group. Language was lower in both the dementia groups compared to the control group. Using a factor analysis revealed that a combination visuospatial orientation and memory could differentiate AD from PDD patients (Figure 4(b)). A score <8 on these parts would indicate an AD type of dementia (<7 if total score is <15).

For the IFS subtests, similar patterns were observed across IFS subtests (Figure 4(c)). Motor programming, conflicting instructions, motor inhibition, inverse digit span, spatial working memory, and abstraction subtests showed lower scores in both dementia groups compared to normal cognition groups. There were no significant differences between Control and PD or between PDD and AD groups, except for verbal inhibition, where the PD group scored lower than the Control group. When trying to create an “AD subscore,” factor analysis revealed the inverse months subtest to be the best option, despite no groups being significantly different from each on this subtest. Consequently, this test was not good at differentiating PDD and AD (Figure 4(d)).

## Discussion

### Main findings

In this study, we assessed the effectiveness of the RUDAS and IFS as brief cognitive assessments for dementia in Lima, Peru, and compared their performance to the MMSE. Both RUDAS and IFS were effective in differentiating dementia groups from normal cognition groups, whereas the MMSE showed a specificity of only 53%. Notably, RUDAS excelled in distinguishing between subtypes of dementia, as evidenced by a RUDAS “AD subscore,” encompassing the visuospatial orientation and memory tasks, perfectly differentiating the AD from PDD groups. IFS also successfully detected subtle cognitive changes in cognitively normal PD patients. Our results highlight RUDAS and IFS as valuable, accessible tools for reducing the time to diagnosis for AD and AD-related dementias, such as PDD. These findings carry important clinical implications and may help direct healthcare policy for Latin American countries and resource-limited settings, where advanced diagnostics are unavailable.

### RUDAS and IFS are effective brief cognitive assessments for AD and PDD

Our study demonstrates that both RUDAS and IFS are effective in differentiating dementia from normal cognition with new insight into their particular relevance in PD, where these tools are less well characterized. RUDAS has been found to be resistant to biases related to education, sex, and language differences, unlike MMSE^32^ and Montreal Cognitive Assessment,^33^ which is further supported by our data. This is important for large and diverse populations. While RUDAS may be associated with age, as found by ourselves and others,^25,34,35^ we found this effect to be fully moderated by dementia presence. This suggests RUDAS may capture accelerated brain aging due to dementia compared to healthy aging. Furthermore, our sub-analysis found that RUDAS was an effective test, even when correcting for age effects. As for IFS, our findings are similar to previous studies on other dementia subtypes which showed a sensitivity of 94.12% and specificity of 94.2% with a cutoff <18 in a Peruvian context,^16^ with similar findings in a Chilean cohort (sensitivity = 0.903; specificity = 0.867).^17^ However, our data supports a higher cutoff of at least <20. We also found no association of IFS with education, and the results of our sub-analysis indicated IFS was robust to age effects.

In contrast to RUDAS and IFS, the MMSE, a widely studied brief cognitive exam in Latin America, exhibited poor performance in distinguishing dementia groups. Other studies also suggest that RUDAS has superior diagnostic accuracy compared to the MMSE and Montreal Cognitive Assessment.^36^ Our findings, consistent with other studies, indicate that a cutoff of 24 provides a sensitivity of 0.818 and specificity of 0.871, while a higher cutoff of 26 yields, which has been recommended by the Movement Disorder Society, provides higher sensitivity (0.967) but lower specificity (0.432).^23^ These findings were consistent with previous literature, which reported low sensitivity (64.1%) and high false positives (15.9%) in Peruvian seniors, particularly those with low literacy.^37^

### RUDAS accurately differentiates PDD from AD

RUDAS was found to be more effective than IFS in distinguishing between PDD and AD, likely since RUDAS probes a broader set of cognitive domains. An “AD subscore,” composed of the visuospatial orientation and memory tasks, perfectly differentiated the PDD from AD groups. Interestingly, for participants with identical total RUDAS scores, the AD subscore did not overlap, suggesting the unique diagnostic value of this scale. This finding underscores the utility of RUDAS in detecting the distinct neuroanatomical changes in AD, such as hippocampal and medial temporal lobe involvement, which contribute to memory impairments.

AD is characterized by memory impairments and alterations in the hippocampus, fornix, and medial temporal lobe circuity.^38^ Consistent with this, AD participants performed worse on the memory subtest compared to PDD participants. Interestingly, AD patients also showed poorer performance on the visuospatial orientation subtest. While RUDAS measures body orientation (e.g., identifying left hand or right foot), the MMSE measures temporal and geographical orientation. However, RUDAS and MMSE orientation tasks were strongly correlated (r = 0.53), and RUDAS visuospatial orientation correlated well with CRAFT 21 recall and MINT (r = 0.62–0.65). Overall, these findings suggest that RUDAS is able to capture classic AD-related changes.

Early stage PDD patients have been found to display broad deficits in attention, executive function, object naming, visuospatial/constructional abilities.^39,40^ Compared to AD, PDD patients have been found to perform worse on attention tasks and better on recognition and recall memory task.^41^ In fact, semantic fluency and visuospatial construction have been found to be important predictors of cognitive decline in PD.^42^ Impairment in these domains may signify more aggressive cognitive deterioration. In contrast, attention deficits are common to most individuals with PD but may not necessarily forebode dementia. However, this work does not investigate conversion or progression to dementia, and future longitudinal studies investigating RUDAS in PDD and AD are necessary. More work needs to be done to identify structural alterations and pathological processes that contribute to these differing profiles in PDD and AD.

### IFS detects subtle cognitive deficits in PD

IFS has also not been well studied in PD, despite the relevance of frontal-striatal network alterations in the disease. Our study found that even normal cognition PD participants performed worse on the IFS compared to controls, suggesting that subtle cognitive changes may be detected early in Parkinson's disease. Furthermore, the verbal inhibition task (i.e., complete the sentence with words that do not make sense in the context) was the only individual task of all brief cognitive assessments that was different between controls and participants with PD. However, other tasks on the IFS were moderately lower in the PD group compared to the control group, although not significant. These findings match other studies that indicate the earliest cognitive deficits are in executive abilities, information-processing speed and working memory, particularly in relation to in set shifting and response inhibition.^39,40,42^ These deficits are believed to be fundamental to the PD pathology due to their relation to motor function^43,44^ and improvement with dopamine replacement therapies.^41,45,46^ One study did indicate that IFS was able to discriminate PD participants with mild cognitive impairment from general mild cognitive impairment participants,^20^ which would further indicate that the frontal deficits are classic to PD. We have previously shown that attention/executive deficits in PD are an early symptom, which tends to plateau early in the disease.^19^ This would be consistent with our findings that PDD and AD did not differ on IFS. Further investigation of the association of IFS within PD and across the course of PD is warranted. Also, linking the IFS exam with structural or functional brain alterations would provide interesting insights into potential disease processes and mechanisms.

### Limitations and future research directions

While this study provides valuable insights into the efficacy of RUDAS and IFS as brief cognitive assessments for dementia subtypes, several limitations should be acknowledged. First, the research did not include patients with mild cognitive impairment or those with other forms of dementia besides AD or PDD, such as vascular dementia. As a result, our findings may not be generalizable to these populations. Future research is needed to determine the effectiveness of these assessments in a broader range of cognitive disorders. Second, the study is cross-sectional, and future work should assess changes in performance over time, particularly as individuals cross important disease boundaries, such as dementia diagnosis or requirement of gait assistance. Additionally, conducting the study at a single site in an urban Peruvian setting may limit the generalizability of the results to broader populations and different healthcare settings. Finally, diagnosis was based on clinical, laboratory, and neuroimaging data, but did not include genetic or pathological data, as well as confirmatory tests investigating amyloid, tau, alpha-synuclein or loss of dopamine-associated transmitter. This presents the possibility of incorrect diagnoses, which may limit the interpretability of results.

## Conclusion

In this study, we have demonstrated the efficacy of RUDAS and IFS as brief cognitive assessments for dementia. Notably, RUDAS proved superior in differentiating between participants with AD and those with PDD, while IFS was effective in capturing early cognitive deficits in PD. These findings underscore the potential of these tools to expedite dementia diagnosis and subtype classification, particularly in resource-limited settings. The findings offer critical insights for clinicians that may lead to more timely and accurate patient care.

## Supplemental Material

sj-docx-1-alr-10.1177_25424823251335193 - Supplemental material for Utility of the Rowland Universal Dementia Assessment Scale and INECO Frontal Screening for differentiating dementia subtypes between Alzheimer's disease and Parkinson's disease dementia Supplemental material, sj-docx-1-alr-10.1177_25424823251335193 for Utility of the Rowland Universal Dementia Assessment Scale and INECO Frontal Screening for differentiating dementia subtypes between Alzheimer's disease and Parkinson's disease dementia by Gregory Brown, Diego Bustamante-Paytan, María Fe Albujar Pereira, Jose Huilca, Katherine Agüero, Graciet Verastegui, Zadith Yauri, Rosa Montesinos and Nilton Custodio in Journal of Alzheimer's Disease Reports
