# Supplementary material for: Utility of the Rowland Universal Dementia Assessment Scale and INECO Frontal Screening for differentiating dementia subtypes between Alzheimer's disease and Parkinson's disease dementia
Source: J Alzheimers Dis Rep. 2025 Apr 17;9:25424823251335193. doi: 10.1177/25424823251335193 (PMC12033470; doi:10.1177/25424823251335193)
Supplement: sj-docx-1-alr-10.1177_25424823251335193 - Supplemental material for Utility of the Rowland Universal Dementia Assessment Scale and INECO Frontal Screening for differentiating dementia subtypes between Alzheimer's disease and Parkinson's disease dementia [file sj-docx-1-alr-10.1177_25424823251335193.docx]

**Supplemental Material**

**Utility of the Rowland Universal Dementia Assessment Scale and INECO Frontal Screening for differentiating dementia subtypes between Alzheimer’s disease and Parkinson’s disease dementia**

**
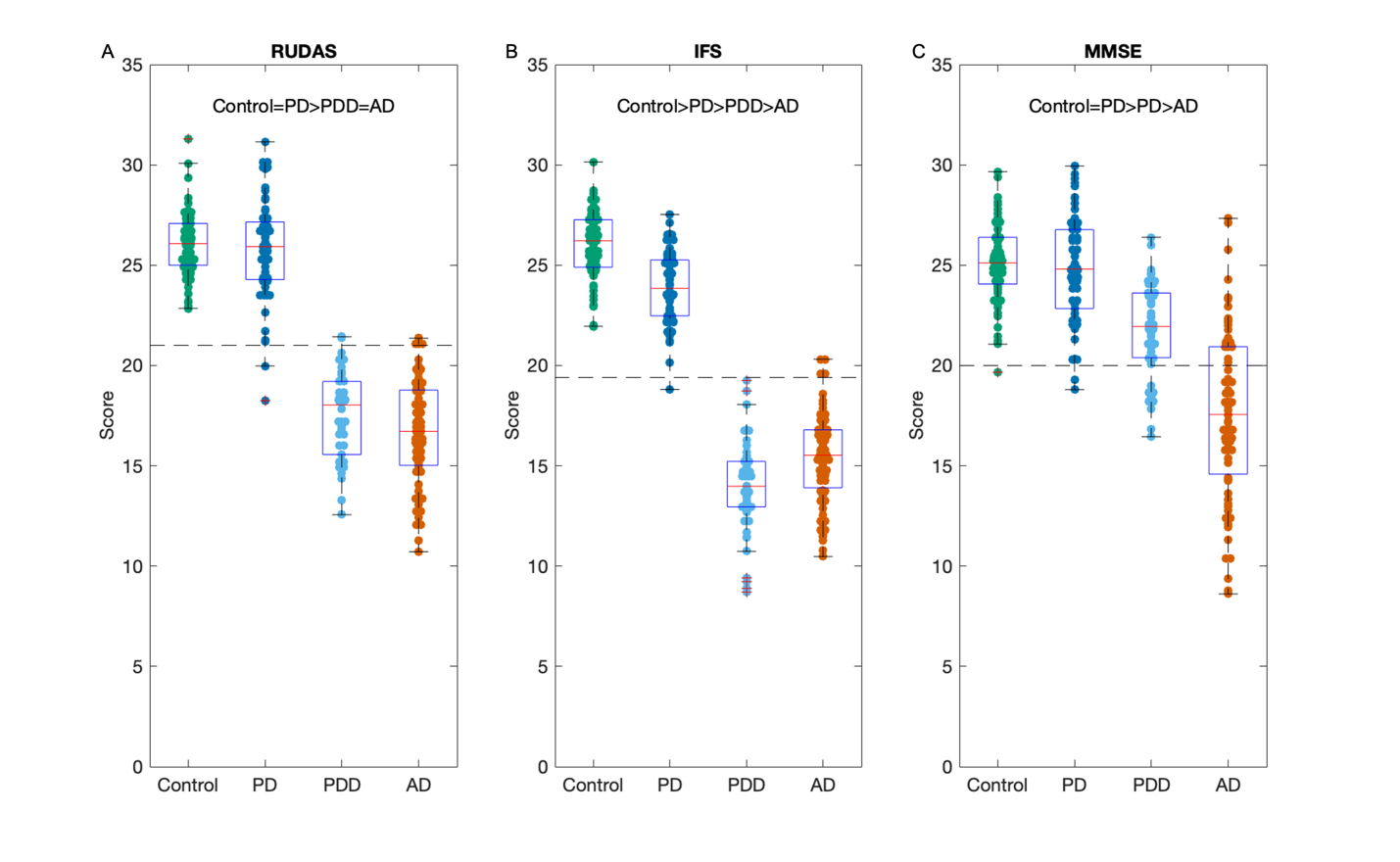
**

**Supplemental Figure 1. Boxplots of brief cognitive tests after correcting for age.** Results for the each of the 3 brief cognitive tests A) Rowland Universal Dementia Assessment Scale (RUDAS), B) Frontal Screening (IFS), and C) Mini-Mental Status Exam (MMSE), after correcting for age to a 70-year-old individual. The data is presented for the four groups: Control, Parkinson’s disease (PD), Parkinson’s disease dementia (PDD), and Alzheimer’s disease (AD). > indicates significant difference between groups for the age corrected metrics. The dotted line represents the optimal dementia cutoff. RUDAS: <21, IFS: <19.4, and MMSE: <20.
